# Posttreatment Surveillance Imaging After Radiation for Head and Neck Cancer

**DOI:** 10.1001/jamanetworkopen.2023.42825

**Published:** 2023-11-10

**Authors:** Allen M. Chen, Jeremy P. Harris, Meng Gan, Rupali Nabar, Tjoson Tjoa, Yarah M. Haidar, Annie Truong, Daniel S. Chow, William B. Armstrong

**Affiliations:** 1 Department of Radiation Oncology, Chao Family Comprehensive Cancer Center, University of California, Irvine, Orange; 2 Department of Internal Medicine, Division of Hematology-Oncology, Chao Family Comprehensive Cancer Center, University of California, Irvine, Orange; 3 Department of Otolaryngology, Chao Family Comprehensive Cancer Center, University of California, Irvine, Orange; 4 Department of Diagnostic Radiology, Chao Family Comprehensive Cancer Center, University of California, Irvine, Orange

## Abstract

**Importance:**

The role of surveillance imaging after treatment for head and neck cancer is controversial and evidence to support decision-making is limited.

**Objective:**

To determine the use of surveillance imaging in asymptomatic patients with head and neck cancer in remission after completion of chemoradiation.

**Design, Setting, and Participants:**

This was a retrospective, comparative effectiveness research review of adult patients who had achieved a complete metabolic response to initial treatment for head and neck cancer as defined by having an unequivocally negative positron emission tomography (PET) scan using the PET response criteria in solid tumors (PERCIST) scale within the first 6 months of completing therapy. The medical records of 501 consecutive patients who completed definitive radiation therapy (with or without chemotherapy) for newly diagnosed squamous cell carcinoma of the head and neck between January 2014 and June 2022 were reviewed.

**Exposure:**

Surveillance imaging was defined as the acquisition of a PET with computed tomography (CT), magnetic resonance imaging (MRI), or CT of the head and neck region in the absence of any clinically suspicious symptoms and/or examination findings. For remaining patients, subsequent surveillance after the achievement of a complete metabolic response to initial therapy was performed on an observational basis in the setting of routine follow-up using history-taking and physical examination, including endoscopy. This expectant approach led to imaging only in the presence of clinically suspicious symptoms and/or physical examination findings.

**Main Outcome and Measures:**

Local-regional control, overall survival, and progression-free survival based on assignment to either the surveillance imaging or expectant management cohort.

**Results:**

This study included 340 patients (mean [SD] age, 59 [10] years; 201 males [59%]; 88 Latino patients [26%]; 145 White patients [43%]) who achieved a complete metabolic response during this period. There was no difference in 3-year local-regional control, overall survival, progression-free survival, or freedom from distant metastasis between patients treated with surveillance imaging vs those treated expectantly.

**Conclusions and Relevance:**

In this comparative effectiveness research, imaging-based surveillance failed to improve outcomes compared with expectant management for patients who were seemingly in remission after completion of primary radiation therapy for head and neck cancer.

## Key Points

**Question:**

Is surveillance imaging for asymptomatic patients in apparent remission from head and neck cancer associated with outcomes?

**Findings:**

In this comparative effectiveness research study of 340 patients, imaging-based surveillance was not associated with improved outcomes compared with expectant management for patients who had achieved a complete metabolic response after completion of primary radiation therapy for head and neck cancer. This comparative effectiveness research suggested that patients who underwent surveillance imaging also experienced a higher incidence of unnecessary procedures and treatment-related complications.

**Meaning:**

These findings suggest that the routine imaging of asymptomatic patients in remission from head and neck cancers should be discouraged; the high rates of false-positive tests were of concern.

## Teaser

This comparative effectiveness research study assesses the use of surveillance imaging in asymptomatic patients with head and neck cancer in remission after completion of chemoradiation.

## Introduction

A considerable proportion of patients treated for head and neck cancer by radiation therapy experience disease recurrence with the likelihood influenced by factors, such as primary site, tumor extent, nodal burden, and histological grade. Despite this, the role of posttreatment surveillance for patients seemingly in remission after successful treatment of head and neck cancer is controversial, and it is unclear how surveillance imaging (ie, routine imaging that is not prompted by clinical symptoms and/or examination findings) in the follow-up setting may be associated with survival. While the use of posttreatment positron emission tomography (PET), typically combined with computed tomography (CT), in the early posttreatment period (usually at 3 months) to guide subsequent management has been well-established for patients treated by primary radiation therapy, the role of additional follow-up imaging is uncertain.^1,2,3^ For instance, among patients with a complete metabolic response to treatment, it is unclear whether subsequent routine surveillance imaging conveys any benefit, and there is currently a lack of evidence to guide clinical decision-making, particularly with respect to cost-effectiveness, in this setting. The purpose of this study was to analyze clinical outcomes, including survival for patients who achieved a complete response to treatment based on initial 3-month PET according to whether patients subsequently underwent imaging-based surveillance or clinically based surveillance.

## Methods

This comparative effectiveness research review was approved by the institutional review board from the University of California, Irvine and informed consent was waived because patient data were deidentified and the study was deemed minimal risk. This study followed the Strengthening the Reporting of Observational Studies in Epidemiology (STROBE) reporting guideline.

### Study Design

The medical records of 501 consecutive patients who completed definitive radiation therapy for newly diagnosed squamous cell carcinoma localized to the head and neck between January 2014 and June 2022 were reviewed to identify patients who achieved a complete metabolic response to initial treatment as defined by an unequivocally negative PET scan using the PET response criteria in solid tumors (PERCIST) scale within the first 6 months of completing therapy.^4^ Cancers of primary cutaneous origin were specifically excluded. Although our institutional standard has traditionally been to acquire a PET with CT (PET/CT) at approximately 3 months after completion of definitive radiation therapy, external circumstances commonly related to logistical considerations (eg, insurance, transportation, etc) contributed to variability in practice patterns regarding the timing of initial posttreatment imaging. The PERCIST scale was selected based on previous studies demonstrating a negative predictive value approaching 100%.^5^ All imaging was reviewed by a board-certified neuroradiologist. When uncertainty or questions arose from imaging findings, patients were presented at a dedicated head-and-neck multidisciplinary tumor conference for further discussion.

### Patients

**Figure 1.**
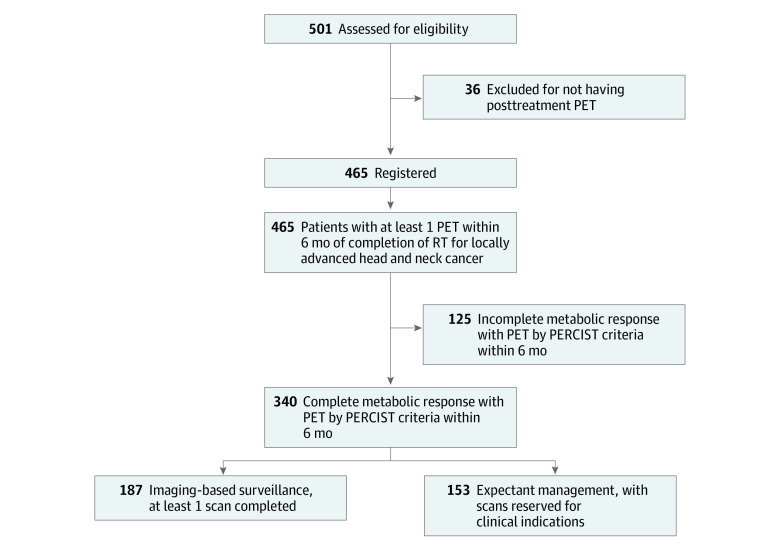
Patient Flow Chart Abbreviations: PET, positron emission tomography; PERCIST, PET response criteria in solid tumors; RT, radiation therapy.

Figure 1 illustrates the flowchart of the 501 patients with localized squamous cell carcinoma of the head and neck, of which 465 (93%) had a PET scan within 6 months after completion of radiation therapy. Among these, a total of 340 patients (71%) achieved a complete metabolic response during this time period and comprised the study population. Notably, 50 of the 340 patients (15%) underwent 2 separate PET scans because the initial posttreatment PET showed an incomplete metabolic response with a decline from initial standardized uptake value maximum but having a value still in excess of 3.0. However, subsequent PET (still within 6-month window) showed achievement of metabolic complete response. One hundred and thirty of the 340 patients (38%) achieved both a complete metabolic response and had radiographic disappearance of all visible gross abnormality based on CT assessment. Of the 340 patients, 203 (60%) received concurrent chemotherapy.

The 340 patients forming the primary study population were then retrospectively stratified into 2 cohorts dependent on whether any additional surveillance imaging was performed in the absence of any clinical symptomology after the documentation of a complete metabolic response to initial therapy. Table 1 outlines the patient and disease characteristics for each cohort.

**Table 1.** Clinical and Disease Characteristics

Characteristic	Patients, No. (%)		*P* value
	Imaging-based (n = 187)	Clinical-based (n = 153)	
Primary site			
Oropharynx	105 (56.1)	85 (55.6)	.22
Larynx or hypopharynx	38 (20.3)	28 (18.3)	
Nasopharynx	30 (16)	19 (12.4)	
Others	14 (7.5)	21 (13.7)	
T-classification			
T0 or T1	53 (28.3)	44 (28.8)	.51
T2	40 (21.4)	39 (25.5)	
T3	50 (26.7)	27 (17.6)	
T4	44 (23.5)	43 (28.1)	
N-classification			
N0	59 (31.6)	45 (29.4)	.56
N1	30 (16)	32 (20.9)	
N2	68 (36.4)	50 (32.7)	
N3	30 (16)	26 (17)	
KPS at diagnosis			
90-100	99 (52.9)	77 (50.3)	.23
80	60 (32.1)	49 (32)	
≤70	28 (15)	27 (17.6)	
Concurrent chemotherapy			
Yes	155 (82.9)	116 (75.8)	.55
No	32 (17.1)	37 (24.2)	
Sex			
Male	139 (74.3)	117 (76.5)	.24
Female	48 (25.7)	36 (23.5)	
HPV status			
Positive	71 (38)	65 (41.2)	.42
Negative	37 (19.8)	41 (26.7)	
Unknown	79 (42.2)	47 (30.7)	

Abbreviations: HPV, human papillomavirus; KPS, Karnofsky performance status.

For the purpose of this analysis, surveillance imaging was broadly defined as the acquisition of a PET/CT, magnetic resonance imaging (MRI), or CT of the head and neck region in the absence of any clinically suspicious symptoms and/or examination findings. Given the absence of any standardized surveillance protocol at our institution during the study, it was expected that variability exist in the type and frequency of imaging. Due to the publication of studies within the last decade attesting to the efficacy of lung cancer screening for smokers, the use of lung CT alone did not qualify as a factor for inclusion in the surveillance cohort.^6^ Patients who embarked on surveillance imaging but developed interval symptoms which prompted imaging were nevertheless categorized into the surveillance imaging cohort. For remaining patients, subsequent surveillance after achievement of a complete metabolic response to initial therapy was performed on an observational basis in the setting of routine follow-up using history-taking and physical examination, including endoscopy. This expectant approach led to directed imaging only in the presence of patient-reported or clinician-elicited symptoms and/or physical examination findings that were clinically suspicious.

Information regarding race and ethnicity was collected from intake forms and considered self-reported. This information was collected based on considerations that head and neck cancer may behave more aggressively in certain populations.

### End Points

Patients were asked to return for follow-up approximately 2 to 3 weeks after completion of radiation therapy and then every 3 months for the first year, 4 to 6 months for the second and third year, and then annually thereafter. The primary end points analyzed were overall survival, progression-free survival, local-regional control, and freedom from distant metastasis.

### Statistical Analysis

Actuarial estimates were determined using the Kaplan-Meier method. Local control was judged to have been attained if there was no evidence of tumor at the primary site based on clinical and radiographic findings at follow-up. Regional failure was recorded separately if there was evidence of a cervical or supraclavicular mass distinct from the primary site. Progression-free survival was defined as survival without evidence of either distant disease and/or local-regional failure. Failure was recorded only if the patient had pathologic evidence of residual disease. Secondary end points included the incidence of second primary cancers, additional biopsies and/or surgical procedures for suspicious findings, and/or complications for subsequent cancer-related procedures. Patient follow-up was reported to the date last seen in clinic or to the date of expiration. All events were measured from the last day of radiation therapy. Median (range) follow-up was 39 (6 to 101) months for the entire patient population and 36 months and 44 months for patients in the imaging-based cohorts and clinically based cohorts. The Wilcoxon rank sum test was used to compare the distribution of time to relapse between RER patients with relapse and SER patients with relapse. Statistical analysis was performed using SAS version 9.4 (SAS Institute). All tests were 2-tailed and statistical significance was set at *P* <.05.

## Results

Among the 340 patients (mean [SD] age, 59 [10] years) included in the study, 201 (59%) were male, 139 (41%) female, 85 (25%) were Asian patients, 22 (6%) were Black patients, 88 (26%) were Latino patients, and 145 (43%) were White patients. A total of 187 patients (55%) underwent imaging-based surveillance, and 153 (45%) were treated expectantly with imaging reserved only in the presence of suspicious symptoms and/or physical examination findings. For those who underwent imaging-based surveillance, the first study consisted of whole-body PET/CT in 130 patients, CT of the head and neck in 32 patients, and MRI in 25 patients. The initial surveillance scan, which was always ordered while patients were asymptomatic, was obtained at a median (range) of 9 (1-50) months after the initial negative posttreatment scan. For patients who underwent imaging-based surveillance, the median (range) number of total scans encompassing the head and neck region that was performed was 4 (1-11). For the 187 patients in the imaging-based surveillance cohort, the number of patients who had a PET/CT, MRI, and CT at any time in the follow-up period (in the absence of symptoms) was 178 (95%), 36 (19%), and 27 (14%), respectively. Among the 153 patients in the clinically based surveillance cohort, the number of patients who had a PET/CT, MRI, and CT at any time in the follow-up period (due to symptoms and/or physical examination findings) was 25 (16%), 8 (5%), and (7%), respectively.

The primary disease site was oropharynx (180 patients); larynx or hypopharynx (66 patients); nasopharynx (49 patients), other (45 patients). The median (range) age was 60 (21-90) years. Additionally , 142 patients (42%) were positive for p16, which was used as a surrogate biomarker for human papillomavirus (HPV).

**Figure 2.**
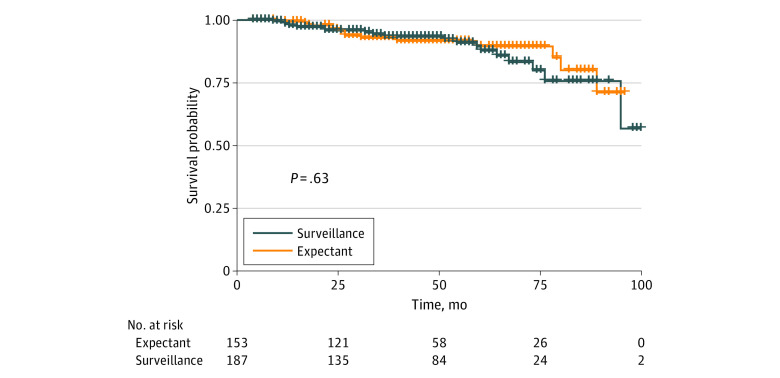
Overall Survival

**Figure 3.**
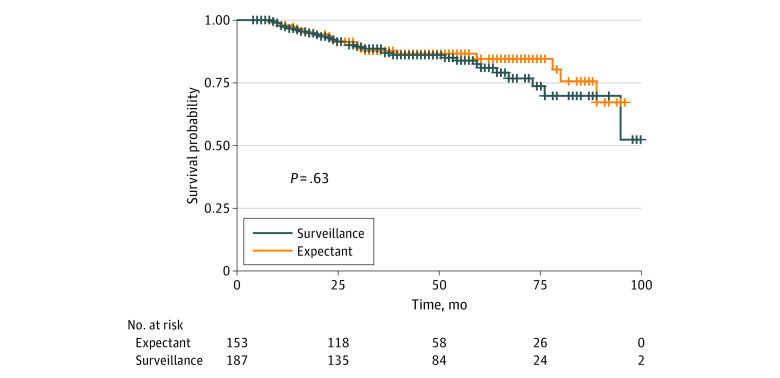
Progression-Free Survival

A total of 51 recurrences (28 local or regional [55%]; 23 distant [45%]) occurred among all 340 patients, 32 (63%) in the imaging-based surveillance group and 19 (37%) in the clinically based surveillance group. There were no differences in the incidence of recurrences according to T-stage or N-stage. Seventeen of the 51 recurrences (33%) in the imaging-based surveillance group were HPV-positive compared with 6 of 19 (32%) in the clinically based surveillance group (*P* = .89). Thirty-nine (77%) and 47 (89%) of 51 recurrences occurred within 2 years and 3 years, respectively, from completion of radiation therapy. There was no difference in 3-year local-regional control (90% vs 93%, *P* = .47), overall survival (94% vs 93%, *P* = .64), progression-free survival (89% vs 88%, *P* = .46), or freedom from distant metastasis (90% vs 90%, *P* = .38) between patients managed by surveillance imaging vs those managed expectantly, respectively. Seventeen of 187 patients in the imaging-based surveillance group developed local-regional recurrence yielding a 3-year local-regional control of 90%. In comparison, 11 of 153 patients in the clinically based surveillance group developed local-regional recurrence yielding a 3-year local-regional control of 93%. Figure 2 and Figure 3 illustrate overall survival and progression-free survival according to whether imaging-based or clinically based surveillance was performed during the follow-up period. Table 2 illustrates subset analysis when stratified by age, performance status, human papillomavirus (HPV), and tobacco use.

**Table 2.** Subset Analysis of Overall Survival by Mode of Surveillance

Factor	Strata, No.	3-y Overall survival by surveillance approach, %		*P* value
		Imaging-based	Clinically based	
Age, y				
≤60	165	94	91	.54
>60	175	89	95	.71
Sex				
Male	201	93	95	.39
Female	139	94	94	.52
T-stage				
T0-T2	176	95	96	.77
T3-T4	164	90	89	.40
Concurrent chemotherapy				
Yes	271	95	94	.83
No	69	88	90	.32
KPS				
90-100	176	94	95	.51
≤80	164	90	89	.38
Primary site				
Oropharynx	190	95	93	.63
Nonoropharynx	150	91	92	.47
Smoking history, PPD				
<50	47	90	90	.33
20-50	69	87	89	.51
0-20	98	91	90	.19
Never	126	95	97	.20
HPV status				
Positive	136	97	96	.52
Negative or unknown	204	90	92	.46

Abbreviations: HPV, human papillomavirus status; KPS, Karnofsky performance status; PPD, packs per day.

Among the 187 patients in the imaging-based surveillance cohort, 84 (45%) underwent a biopsy (41 head and neck; 20 lung or mediastinum; 15 abdomen or pelvis; 8 other) at any point during the follow-up period with 41 having pathological evidence of cancer, leading to a positive predictive value of 49%. The most common nonmalignant findings diagnosed on biopsy in this cohort were in-field radiation fibrosis and/or necrosis (13 patients [30%]), reactive nodal adenopathy (8 patients [19%]), nonspecific pulmonary nodule including granuloma, aspiration, pneumonia, chronic inflammation (8 patients [19%]), thyroid nodule (5 patients [12%]), benign salivary tumor (4 patients [9%]), mucosal hyperplasia/sinusitis (3 patients [7%]), hepatic or pancreatic cyst (2 patients [5%]), benign adrenal lesion (2 patients [5%]), bony island/enostosis (2 patients [5%]), and colon adenoma (2 patients [5%]). Notably, 21 patients (11%) in the imaging-based surveillance cohort underwent more than 1 biopsy during the follow-up period. In comparison, 37 of the 153 patients (24%) in the clinically based surveillance cohort underwent biopsy at any point during the follow-up period with 33 (89%) having pathological evidence of cancer, yielding a positive predictive value of 89%.

The proportion of patients who underwent any open surgical procedure thought to be associated with cancer and/or its evaluation in the follow-up period was 15% and 5% in the imaging-based and clinically based cohorts, respectively. The corresponding proportion of patients who underwent neck dissection was 9% and 2%, respectively. The incidence of osteoradionecrosis, fistula formation, and subsequent procedures and/or complications requiring tracheostomy was significantly higher among patients in the imaging-based vs clinically based surveillance cohorts (9% vs 5%, *P* = .01). A total of 68 histologically-proven second malignant neoplasms of new primary origin were diagnosed at a median (range) of 20 (3-73) months: 15 (68%) skin; 11 (16%) lung; 9 (13%) other head and neck (including thyroid and salivary gland cancer); 9 (13%) genitourinary; 6 (9%) esophageal or gastric, 5 (7%) breast, 5 (7%) hepatobiliary, 3 (4%) hematologic, 3 (4%) brain, and 2 (3%) soft tissue sarcoma. Eleven patients (16%) developed separate third primary cancers. There was no difference in the diagnosis of second malignant neoplasm between patients assigned to the imaging-based and clinically based surveillance cohorts.

## Discussion

Routine imaging is performed for asymptomatic patients who seem to be in remission from many types of cancer to detect relapse, under the presumption of an improved probability of salvage. The overall rationale behind surveillance is to detect recurrences early with enough sensitivity and specificity so that morbidity and mortality can be reduced. While it may seem intuitive that more aggressive and frequent imaging will lead to improvements in outcome, this issue is not as straightforward.

The results of the present study, while seemingly paradoxical, are consistent with those of others which have failed to demonstrate a benefit to surveillance imaging among patients who have successfully completed treatment for head and neck cancer. Ho et al^7^ analyzed the impact of 12-month and 24-month PET/CT surveillance and showed no survival advantage for the subset of patients with a negative PET/CT at 3 months posttreatment. Similar to the results of the present study, only 6% of PET/CT scans which were deemed equivocal were found to have cancer. Koshkareva et al^8^ also demonstrated that recurrences after an initial posttherapy PET/CT that was negative were generally rare, especially for patients with HPV, who could likely be spared additional routine imaging surveillance. In the only prospective study evaluating the potential role of PET-based surveillance to detect recurrence of head and neck cancer, Perie et al^9^ showed that systematic imaging performed routinely at 1 year in the absence of symptoms was associated with a much higher incidence of subsequent futile procedures compared with when imaging was reserved for cases motivated by clinical suspicion. In a study of 1508 asymptomatic patients without evidence of disease and without clinically suspicious findings at 1-year post-treatment, Ping et al^10^ showed that surveillance imaging had low yield, high cost, and failed to impact survival. Beswick et al^11^ also reported that 79% and 95% of asymptomatic recurrences from head and neck cancer occurred within the first 12 and 24 months, respectively, after completion of definitive chemoradiation, suggesting that routine surveillance thereafter is of limited use.

Our findings that relapses were equally well detected by changes in symptoms and physical examination findings when compared with routine imaging in asymptomatic patients emphasizes the importance of compliance to clinical follow-up. Indeed, the diagnostic sensitivity of direct endoscopy for visualizing the pharyngeal axis has been demonstrated to be excellent.^12,13^ In a prospective study of 22 patients with newly diagnosed head and neck cancer, Laubenbacher et al^12^ showed the endoscopy was more accurate than either MRI or PET in the staging of primary head and neck cancers. Moreover, Keller et al^13^ showed that 4 of 10 primary tumors detected initially by endoscopy were not identified with PET and 4 of 18 primary tumors were not found with PET/CT. While other studies have suggested that clinical examination and endoscopy can be replaced by imaging in the evaluation of head and neck cancer, the results of the present study argue to the contrary and emphasize that the diagnostic accuracy of many imaging studies still limits its use in the surveillance setting.^14^

The high incidence of false-positive findings, occurring both within and outside the previous field of radiation, among patients who underwent surveillance imaging was noteworthy, and the resultant impact on quality of life must also be considered, especially since all these patients were asymptomatic. It has been well established that the effects of previous treatment, such as surgery, radiation, and chemotherapy, can distort normal anatomical landmarks and induce inflammation and infection, which can be misinterpreted as disease on imaging.^15^ The head and neck, in particular, is impacted by phenomenon, such as tumor regression and alterations in muscle density, fat distribution, subcutaneous edema, and fluid shift, during and after treatment, which can lead to diagnostic challenges. A significant number of incidental findings—those that were diagnosed outside of the radiation field and unrelated to the indication of the imaging study—were also detected, a high proportion of which were subsequently worked up. Although a proportion of these were already present at the time of initial treatment, these findings contributed to decision-making dilemmas in the follow-up setting. Britt et al^16^ similarly showed that greater than one-third of all patients with head and neck cancer had incidental findings on PET/CT at diagnosis, most of which required additional evaluation and were benign.

The underlying reasons for why surveillance imaging continues to be performed are speculative but may be associated with external factors, such as pressure from peers and supervisors, the convenience with which a test can be ordered, demands by the patient and/or family, and the desire to avoid malpractice claims. Notably, the detection of a radiographic abnormality may produce a cascade effect potentially leading to unnecessary invasive procedures (including ironically, in some cases, additional imaging surveillance), increased costs, and undue patient anxiety for a finding that is ultimately benign. Indeed, in the present study, the use of biopsy because of surveillance imaging with pathology subsequently showing fibrosis or necrotic tissue was the most common source of false-positive findings. More importantly, the effects of radiation on impaired wound healing after biopsy led to the precipitation of complications, such as osteoradionecrosis, fistula formation, and tracheostomy, as the result of manipulation and/or trauma of previously irradiated tissue.^17^ Cheung et al^18^ similarly showed that 50% of patients who had false-positive posttreatment PET/CT scans who underwent invasive procedures experienced significant morbidity. Additionally, the exposure risks from imaging are cumulative as ionizing radiation carry the theoretic harm of radiation-induced carcinogenesis

The potential for lead time bias needs to be acknowledged, as well, in any surveillance program. This is especially germane in the setting of head and neck cancer as the prognosis for locally recurrent and/or metastatic head and neck cancer is poor, and it is controversial how much more curable early (presumably low-volume) recurrences may be compared with those that might be detected later.^19^ While tumor bulk has generally been accepted as being of prognostic significance for patients undergoing re-irradiation for recurrent head and neck cancers, a multitude of other factors, such as interval between courses, location of recurrence, and performance status have been shown to be of equal, if not, greater importance.^20,21,22^ For patients with distant metastatic disease, the median overall survival has generally been estimated at approximately 12 months with most patients dying by 24 months.^23^ Indeed, others have shown no difference in survival between imaging-detected recurrences and those found by physical examination.^7^

For elderly patients and other patients who may not be able to tolerate the rigors of traditional salvage therapy, including surgery, re-irradiation, and/or chemotherapy, the use of early detection must especially be questioned. Patients with head and neck cancer have been shown to have significant competing risks of death, which may confound any benefit to early detection of recurrence.^24^ Despite the suggestion that younger, healthier patients (eg, those with HPV) may seemingly derive the most gain from surveillance due to increased life expectancy and possibly an atypical pattern of recurrence, we were nonetheless unable to identify any subset of patients that benefited from such a strategy.^25^ Of further interest was the fact that no difference was observed in the detection of second primary cancers (nonpulmonary) between the cohorts, which was not surprising given that screening in high-risk populations has never been shown to be effective, outside of the use of CT for lung cancer.^6^

### Limitations

This study has limitations. This was a nonrandomized study that selected for patients with a relatively favorable prognosis from the onset. Kao et al^26^ showed that 2-year overall survival was dramatically different (100% vs 32%) for patients with a negative vs positive PET/CT result within 6 months of completing radiation therapy. Furthermore, we could not ascertain whether each patient was truly asymptomatic for inclusion into this study because some undoubtedly had evidence of treatment-related sequelae. Additionally, the decision of whether to perform surveillance imaging was arbitrary and made on individualized basis. In other words, no dedicated policy was in place to guide the acquisition of further imaging after the initial PET/CT was performed. While there were no significant differences in the distribution of characteristics between patients who underwent surveillance-based vs expectant management, the role of physician- and patient-related biases still must be recognized. These potentially include factors associated with the underlying disease, as well as socioeconomic considerations. Because central review of imaging was not performed, we could not control for any technical variabilities in how images may have been acquired and/or interpreted. Although it was challenging in some cases to discern between true recurrences and second primary cancers (eg, solitary lung lesion) for classification purposes, there were no differences between the patients who received imaging-based surveillance and an expectant approach with respect to any of the survival end points.

Last, the heterogenous patient population makes drawing definitive conclusions inherently difficult. Given the relatively small sample size and limited number of events, it was impossible to determine whether specific cohorts might derive more benefit from surveillance than others. For instance, it is possible that small, low-volume tumors, particularly those associated with HPV, might not require the same degree of surveillance as higher-volume, HPV-negative cases. As such, HPV status was unknown in many cases, which was a major limitation. HPV status is not routinely obtained for tumors arising from nonoropharyngeal subsites given the lack of consensus regarding its associated prognostic significance.^27,28^ Furthermore, this study, like many others, considered p16-positivity to be equivalent to HPV-positivity. However, it is now established that patients with p16-positive and HPV-negative squamous cell oropharyngeal carcinomas do not have the same favorable prognosis as those with p16-positive and HPV-positive tumors. Instead, the prognosis is intermediate for patients with p16-positive and HPV-positive and p16-negative and HPV-negative cancer, which suggests that confirmatory testing for HPV is necessary.^29^

## Conclusion

These findings suggest that imaging-based surveillance failed to improve outcomes for patients who appeared to be in remission after completing primary radiation therapy for head and neck cancer. While the use of imaging in the context of clinical suspicion has been shown to be valuable, the routine acquisition of surveillance imaging in asymptomatic patients should be discouraged. This assertion is consistent with published guidelines^30,31,32,33^ recommending follow-up imaging after the initial posttreatment baseline only if the patient displays worrisome or equivocal signs or symptoms. While a discussion regarding the implications with respect to cost-effectiveness lies beyond the scope of this work and has been reported by others,^33^ most patients in this study underwent routine surveillance for no apparent benefit. Additional studies that take into account the probability of recurrence, the distribution of metastatic disease, and the availability of effective therapies for recurrent disease are needed to gauge its true effectiveness. Larger, multi-institutional studies are needed to further evaluate our findings.

## Notes

Supplement.
Data Sharing Statement
