# Supplementary material for: Posttreatment Surveillance Imaging After Radiation for Head and Neck Cancer
Source: JAMA Netw Open. 2023 Nov 10;6(11):e2342825. doi: 10.1001/jamanetworkopen.2023.42825 (PMC10638654; doi:10.1001/jamanetworkopen.2023.42825)
Supplement: Supplement. — Data Sharing Statement [file jamanetwopen-e2342825-s001.pdf]

Data Sharing Statement

Chen. Posttreatment Surveillance Imaging After Radiation for Head and Neck Cancer. *JAMA Netw Open*. Published November 10, 2023. doi:10.1001/jamanetworkopen.2023.42825

Data
Data available: No

Additional Information
Explanation for why data not available: Data available in anonymous de-identified fashion upon request
